# Notes and News

**Published:** 1889-06-29

**Authors:** 


### Notes and News.

Collected and Communicated.

The working men of Coventry have secured their wish of being better represented on the managing board of the hospital, and it now befits them to choose carefully the best of their members to take the new duties. There are 450 governors of Coventry Hospital, but only 80 took the trouble to vote. The working men got a majority of 24.

The 15th of July is being eagerly expected at the London Hospital, and also at the Medical College. On that date the Duke of Cambridge will distribute the prizes to the students and nursing probationers, and will also present (in the name of the officials and friends of the hospital), Sir Andrew Clark with his portrait painted by the late Frank Holl. During the many years Sir Andrew Clark was senior physician at the London he always had a large following of respectful pupils, and was a great favourite. A replica of the portrait will be hung in the library of the Medical College.

On June 19th was laid the foundation-stone of Dufftown Cottage Hospital, presented by Sir George Stephen, Bart., Montreal, to the parishes of Mortlach and Glenrinnes. When Sir George Stephen paid a visit to his native glen last summer he presented the handsome sum of £5,000 for the purpose of erecting, equipping, and endowing this building for the sick poor. In the carrying out of the scheme no time has been lost, for already the mason work of the structure is well forward. The plans were drawn by Messrs. A. and W. Reid, architects, Elgin. The site of the new hospital, which was generously given by Lord Fife at a nominal feuduty, and which is on the Tininvie lands, is a charming one. The frontage of the building looks up the picturesque and historically-interesting valley of the Dullan, the slopes of which are now richly wooded.

It is a pity that public interest cannot be roused in any medical subject without quickly developing into a scare. There are two scares on in London just now, leprosy and hydrophobia. It is proposed to raise a threefold memorial to Father Damien—first, a monument at Molokai; second, a leper ward in the London Hospital; and third, an enquiry into the cause and cure of leprosy. The second proposition is open to objection, because once there is a ward lepers from all places will congregate there, and, probably, instead of two or three lepers in London we shall have a dozen or more. At present an ordinary isolation ward is found convenient for any leper needing treatment. It is stated that Father Conrady, now priest at Molokai, has contracted leprosy. On Sunday afternoon Mr. Clifford is going to deliver his lecture on Father Damien at Toynbee Hall.

The Convalescent Home at Morthoe, built by Mr. Rocke and his sister Mrs. Payne as an adjunct to the North Devon Infirmary, was formally opened on June 15th. The site of the home has been well selected, and covers about an acre and a half of ground which was generously given free of cost by Lord Fortescue. It is well protected from cold winds, and still commands an admirable view of the bay, with Lundy Island directly in front. Mr. A. Lander was the architect, and Mrs. Crang, of Barnstaple, has been appointed matron.

The will of the late Mr. John Richardson, of Rochdale-road, Manchester, pawnbroker, watchmaker, silversmith, and jeweller, has been proved at the Manchester District Registry. He has left £15,000 to public institutions, including £2,000 each to the Manchester Infirmary and Boys' and Girls' Refuges; £1,000 each to St. Mary's Hospital, Salford and Pendleton Hospital, General Hospital for Sick Children, Henshaw's Blind Asylum, the Lock Hospital, the Ladies' Jubilee School for Orphans, the Hospital for Incurables at Mauldeth, and the Ardwick Green Industrial Schools.

The friendly societies of Ripon held their annual demonstration on Sunday, June 9th, when the Dean preached in the Cathedral. In making an appeal for the Ripon hospitals, the Dean alluded first to the Cottage Hospital. When last he addressed them, it was a dispensary and it remained so still. The old privileges, and they were very great, still continued—they were untouched. But the house had been utilised as a Cottage Hospital. The collection at the close of the service amounted to £17. In the afternoon a procession went to the Drill Hall, where a short address was delivered by the Mayor.

The committee appointed at the annual meeting of Governors of Halifax Infirmary have given careful and anxious consideration to the subject of the overcrowded state of the infirmary, and the measures which they think it necessary to take. The matters referred to the committee have proved to be of so serious and important a nature, that it is considered advisable to issue the report in printed form, which report we have received and read with interest. The committee have worked very hard and have been munificently supported by their friends. The whole story of hospital work can scarcely beat the generosity and energy lately displayed at Halifax, which will shortly result in a new infirmary on a new site.

A correspondent of the *Torquay Directory* tells the story of the Moretonhampstead Convalescent Home. The late Dr. Lovell Phillips, who for many years resided at Torville, was in the habit of spending a portion of the summer at Moretonhampstead, and he was so much benefited by the bracing air that he often expressed the desire that some scheme should be devised by which the sick poor might be enabled to benefit by a few weeks' residence on the Moors. When Dr. Lovell Phillips died, his two daughters endeavoured to give expression to their father's wish by providing a small institution for the benefit of the poor, and which should be at the same time a kind of memorial to their parent. Accordingly, a little over thirteen years ago, these ladies took a large country house at Moretonhampstead, situated in the main street, surrounded by high walls, and having extensive gardens. Eight years ago a modest-sized wing was added, so that at the present time the home is capable of accommodating twenty-two persons—eight men and fourteen women. The entire establishment is under the personal supervision of the Misses Lovell Phillips, assisted by a resident matron and a general servant, the inmates affording ready help whenever needed. The home is open for nine months in the year, commencing on April 1.

The following vacancies are announced this week, the amount of the salary in each case being given after the name of the institution:

**Resident Medical Officers.**

Manchester Consumption Hospital. Medical Officer.—£60, board, &c.
The London Throat Hospital. House Surgeon.—£50, board, &c.
Hospital for Diseases of the Throat, Golden-square. Medical Officer.—£50, board, &c.
Southampton Infirmary. Assistant House Surgeon.—Board, &c.
Bury Dispensary Hospital. Junior House Surgeon.—£60, board, &c.
Derbyshire General Infirmary. Assistant House Surgeon.—£10, board, &c.
St. Luke's Hospital. Clinical Assistant.—Board, &c.
East London Hospital for Children. House Physician.—Board, &c.

**Non-Resident Medical Officers.**

Haverstock Hill Provident Dispensary. Medical officer.
Paddington Vestry. Two Inspectors of Nuisances.—£120.
Charing Cross Hospital. Assistant Anæsthetist.
St. Mary's Hospital Medical School. Demonstrator of Anatomy.
The Hospital for Women, Soho-square. Clinical Assistants.
King's College, London. Professor of Dental Surgery.

---

We welcome the tenth annual report of the Boys' Orphanage, Blackheath Hill, with extra pleasure, because that institution is conducted on non-voting principles. There are at present 47 boys in the school, and they are all taught trades and given a start in life. The expenses last year reached £1,096.

---

The Sanitary Hospital at Muswell Hill was formally opened on June 15 by Alderman Morton Lathom, J.P. About seventy guests were present, and the matron, Mrs. Newton, dispensed tea and coffee from the administrative building. All the arrangements of the hospital were greatly admired, and there can be no doubt that it will do good work.

---

On St. John the Baptist's Day, the members and associates of the Grand Priory of the Order of the Hospital of St. John of Jerusalem in England held their annual general assembly, which was numerously attended, at the Chancery of the Order, St. John's-gate, Clerkenwell. By permission of the Queen—the Sovereign Head and Patron of the Order—the commemoration services were held in the morning at the Chapel Royal, Savoy. The sermon was preached by the Right Rev. the Bishop of Gibraltar, chaplain of the Order.

---

At the annual meeting of the Montrose Asylum and Infirmary Board, Mr. James M. Ross, convener of the House Committee of the asylum, submitted the annual report. It showed that the population of the house had remained almost unchanged during the year—the number being 500 this year against 501 last year. Owing to some changes relative to private and pauper patients, there had been a diminution of the revenue of £428 odd, leaving a balance of £1,204, which was smaller than the balance at the end of previous year. Notwithstanding the inclement season of 1888, the farm and garden had done well. The new hospital was rapidly advancing towards completion, but a great deal remained to be done internally. The report, after referring to the healthful employment afforded to the patients in connection with the hospital, concluded by thanking the medical officers, matron, nurses, etc., for the efficient manner in which they had discharged their duties and the ladies and gentlemen who had gratuitously given their services for concerts and theatrical entertainments.

Hospital Saturday at Sunderland resulted in over £200, against £186 last year. The fund has expressed its regret that Mr. Webster, their chairman, is leaving them to become one of the secretaries of the London Hospital Saturday Fund. The ladies did good service at Sunderland as collectors, and were heartily thanked at the meeting held on Saturday last.

---

The new hospital at Dover which is being erected in commemoration of the Queen's Jubilee, and is rapidly approaching completion, had a narrow escape from destruction by fire lately. A woman, who had been tramping through the district and had taken shelter in the building, lit a fire in the corner of the basement. She then went to sleep, leaving the burning mass, which was dangerously near a stack of timber and other inflammable material. The glare was noticed by the occupants of an adjoining house, with the result that the police appeared upon the scene, extinguished the fire, and took the woman into custody.

---

From Adelaide, Australia, distant 12,000 miles from London, the secretary of the Seashell Mission, 27, Benedict-road, S.W., has received a most beautiful collection of shells, gathered by the members of the Ministering Children's League, the first branch of which, in Adelaide, was established by Mrs. W. Storrie, of that place, who is now in London, and brought the shells with her from Australia for the mission. Our readers may be glad to be reminded that the Countess of Meath, 83, Lancaster-gate, W., is the president of the Ministering Children's League. Her Serene Highness the Princess Victoria Mary of Teck has just become president of the Seashell Mission.

---

So far as can yet be told the Hospital Sunday Fund is larger this year than ever. St. Matthias, Earl's Court, has been beaten, though the collection there was very large, and St. Michael's, Chester-square, heads the list with a total of over £1,000. Amongst other large amounts already noted are St. Paul's Cathedral, the three services from which produced £237; St. Mark, North Audley-street, £210 12s. 3d.; St. Stephen's, South Dulwich, £166; Union Chapel, Islington, £105 0s. 8d.; St. James's, West Hampstead, £101 5s.; St. Paul's, Forest Hill, £90 4s. 2d.; St. Paul's Presbyterian Church, Westbourne-grove, £85 12s. 7d.; St. Peter's Brockley-hill, £82 13s.; St. Matthew's, Brixton, £82 5s. 2d.; and Holy Trinity, Tulse Hill, £82. Among the private contributors are the Marquis of Salisbury, £100; D. C., £200; Mr. Ludwig Mond, £100; A. A. C., £100; Mr. John Smith, £100; and Lady Tilson, £50.

---

At Eccleston-square Church, Belgrave-road, the Rev. J. Hiles Hitchens, D.D., preached last Sunday on behalf of the hospitals. He chose as his text in the morning Matthew xxi., 14: "And the blind and the lame came to Him in the Temple, and He healed them." Dr. Hitchens pointed out that Christ's great object was to save men's souls, but that He not infrequently cured men's bodies of disease that He may thereby prepare the sufferer's mind for the reception of Divine truth. This thought was enlarged and illustrated. The words of a missionary to the Chinese being quoted: "The Chinese cannot immediately understand Christianity in the Church, where the teaching is spiritual or controversial; but they can understand Christianity in the hospital, where suffering and helplessness make us brothers and sisters in wretchedness and in prayer for relief." Dr. Hiles Hitchens closed by entreating his hearers to imitate Christ in seeking the physical welfare of their fellow-men. There was a large congregation, and the collection surpassed that of the previous year.

